# The clinical outcomes of selenium supplementation on critically ill patients: A meta-analysis of randomized controlled trials

**DOI:** 10.1097/MD.0000000000015473

**Published:** 2019-05-17

**Authors:** Yan Zhao, Mengmeng Yang, Zhi Mao, Rui Yuan, Li Wang, Xin Hu, Feihu Zhou, Hongjun Kang

**Affiliations:** Department of Critical Care Medicine, Chinese PLA General Hospital, Beijing, China.

**Keywords:** meta-analysis, randomized controlled trials, selenium supplementation, trial sequential analysis (TSA)

## Abstract

**Purpose:**

Selenium supplementation is a potentially promising adjunctive therapy for critically ill patients, but the results are controversy among studies. Accordingly, we performed this meta-analysis to more clearly detect the efficacy and safety of selenium supplementation on critically ill patients.

**Methods:**

Systematic literature retrieval was carried out to obtain RCTs on selenium supplementation for critically ill patients up to August 2017. Data extraction and quality evaluation of these studies were performed by 2 investigators. Statistical analyses was performed by RevMan 5.3. Trial sequential analysis (TSA) was conducted to control the risks of type I and type II errors and calculate required information size (RIS).

**Results:**

Totally 19 RCTs involving 3341 critically ill patients were carried out in which 1694 participates were in the selenium supplementation group, and 1647 in the control. The aggregated results suggested that compared with the control, intravenous selenium supplement as a single therapy could decrease the total mortality (*RR* = 0.86, 95% CI: 0.78–0.95, *P* = .002, TSA-adjusted 95% CI = 0.77–0.96, RIS = 4108, n = 3297) and may shorten the length of stay in hospital (MD −2.30, 95% CI −4.03 to −0.57, *P* = .009), but had no significant treatment effect on 28-days mortality (*RR* = 0.96, 95% CI: 0.85–1.09, *P* = .54) and could not shorten the length of ICU stay (MD −0.15, 95% CI −1.68 to 1.38, *P* = .84) in critically ill patients. Our results also showed that selenium supplementation did not increase incidence of drug-induced side effect compared with the control (RR 1.04, 95% CI 0.83 to 1.30, *P* = .73).

**Conclusions:**

The current evidence suggests that the use of selenium could reduce the total mortality, and TSA results showed that our outcome is reliable and no more randomized controlled trials are needed. But selenium supplementation might have no effect on reducing 28-days mortality as well as the incidence of new infections, or on length of stay in ICU or mechanical ventilation. However, the results should be used carefully because of potential limitations.

## Abstract 2

Supplemental Digital Content is available in the text

## 1 Introduction

Endoplasmic reticulum stress, oxidative stress and inflammatory response are increasingly being recognized as the central pathophysiology for critically ill patients. Especially the development of sepsis, septic shock, and multiple organ failure is responsible for a longer hospitalization period and increased risk of mortality.^[1,2]^ Previous studies indicated that the circulating antioxidant and anti-inflammatory levels would decrease rapidly after injury, sepsis, or surgery and would remain below the normal levels for several days or even weeks.^[3]^ The severer the trauma, the systemic inflammatory response syndrome (SIRS), or the sepsis, the larger the depletion of antioxidants appears to be.^[4]^ These changes are associated with an increase in the free radical generation, an augmentation of the systemic inflammatory response, and are playing a direct role in cell death, increased morbidity, and even higher mortality in the critically ill patients.^[3–5]^ Also, studies have proved that special enzymes such as superoxide dismutase, catalase, and glutathione peroxidase (including their cofactors such as selenium, zinc, iron, and manganese), sulfhydryl group donors (glutathione), and vitamins (vitamins C, E, and β-carotene) can form a functional network to protect physiological body from the above injury mechanisms. Current studies all focus on nutrition support with these compositions that may play a critical role in the recovery of the critically ill patients.

Selenium, a trace element, is one of the essential nutrients with regulatory, immunologic, and antioxidant functions. It may play an important role as an antioxidant as well as an anti-inflammatory in the glutathione peroxidase system.^[6]^ Supplementation of selenium is a promising adjunctive therapy for patients with SIRS, sepsis, or septic shock.^[7]^ Up to now, many clinical trials have studied the effect of selenium, being administered intravenously as a monotherapy, on clinical outcomes of critically ill patients (such as mortality, the length of ICU stay, the length of hospital stay, new infections). However, most of these current studies were performed in relatively small patient populations with trauma, SIRS, or sepsis, which are underpowered to detect the treatment effect on clinically outcomes. More importantly, the results are controversial between each other. More recently, several meta-analyses have been performed about selenium supplement on critically ill patients. In 2015, the meta-analysis of Allingstrup et al^[8]^ demonstrated that selenium supplement can reduce the overall mortality of critically ill patients. However, in 2016, Manzanares et al^[9]^ reported that selenium therapy could not reduce the mortality and improve other clinical outcomes of critically ill patients. In consideration of these inconsistencies, we carried out this meta-analysis of the randomized controlled trials (RCTs), aiming to detect the efficacy and safety of selenium supplementation on critically ill patients more clearly.

## 2 Materials and methods

### 2.1 Protocol and registration

This meta-analysis of randomized controlled trials was performed according to the PRISMA (Preferred Reporting Items for Systematic reviews and meta-analyses) recommendations. A protocol for this meta-analysis has been registered on PROSPERO (http://www.crd.york.ac.uk/prospero) and the registration number is: CRD42017079365.

### 2.2 Literature search

Three search engines, namely PubMed (1966–2017.8), Embase (1974–2017.8), and Cochrane library (Issue 8, 2017) were retrieved. The following key words were used: 'selenium’, 'selenium derivative’, 'selenious acid’, 'sodium selenite’, ’antioxidant cocktails’, 'selenium compounds’, ’randomized controlled trial’, ’randomized’, ’randomly’, ’trial’, ’clinical trials’, ’controlled clinical’, ss"[Mesh], ‘clinic. No limit was set in the process. In addition, the references listed at the end of the paper were also manually checked to filter potentially eligible researches.

### 2.3 Inclusion and exclusion criteria

1. Trials: RCTs only, including information about random sequence generation, allocation concealment, and blinding method.
2. Participants: All the critically ill patients included in the studies were suffering the following diseases: SIRS, sepsis, septic shock, acute pancreatitis, multiple organ failure or severe multiple injury, and so on.
3. Interventions: The patients were randomly allocated to the selenium supplementation group or the control according to the telephone computer system or computerized randomization or random number table. For the selenium supplementation group they were given parenteral selenium supplementation singly at different doses (not in combination with other antioxidant micronutrients), while the control were given placebo or maintenance dose selenium or no intervention. In addition, critical patients in the 2 groups could receive other treatment.
4. Outcomes: Primary end points: mortality at day 28 and total mortality (regardless of the follow-up period). Secondary end points: new infection, length of stay in ICU, length of stay in hospital and length of mechanical ventilation during follow-up.

### 2.4 Data extraction

According to Table 1, 2 investigators (Yan Zhao and Hongjun Kang) independently read the titles, abstracts and full texts with the following procedures:

1. examining titles and abstracts to remove obviously irrelevant studies,
2. retrieving the full texts of potentially relevant trials,
3. examining full texts for compliance of studies with eligibility criteria, and
4. making final decisions on data entry and proceeding to data collection.

**Table 1:**
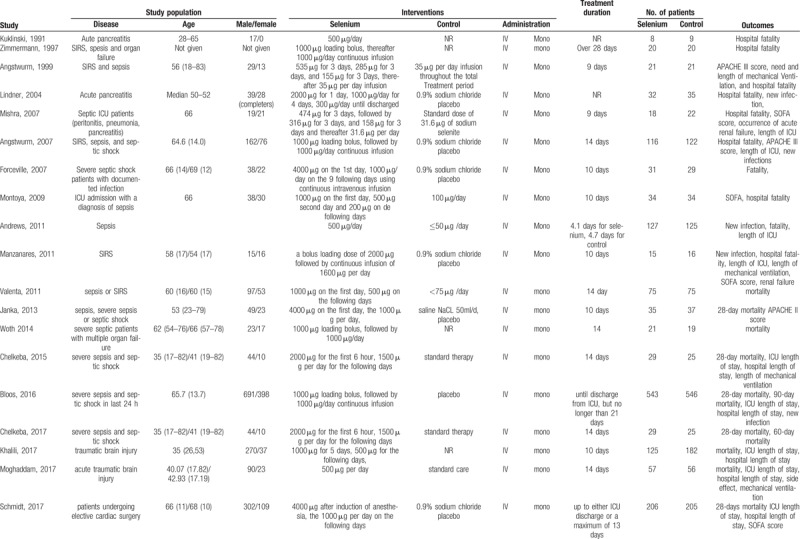
Characteristics of included trials.

Patient's baseline information (treatment strategy, dose, and duration of supplementation) and detailed methods of research design (publication year, research settings, designs, methods of randomization, allocation concealment, blinding) were extracted from the selected studies. Disagreement was solved by discussion with the third investigator (Feihu Zhou).

### 2.5 Quality evaluation

Each study assessed the methodological qualities of trials by 2 investigators (Yan Zhao and Hongjun Kang) independently. The criterion was based on criteria described in Cochrane Reviewer's Handbook 5.1.0, including the following risk of selection, performance, detection, attrition and reporting bias domains: random sequence generation, allocation concealment, blinding, incomplete outcome data, intention to treat analysis.

### 2.6 Data synthesis and statistical analysis

Differences were calculated as risk ratios (RRs) and expressed with 95% confidence intervals (CIs) for dichotomous outcomes and mean differences (MDs) with 95% CIs for continuous outcomes. Heterogeneity across analysis was done using *I*^2^ statistic, which is a quantitative measure of the inconsistency of the across analysis. Studies with an *I*^2^ statistic of 25% to 50%, 50% to 75%, and >75% are considered as low heterogeneity, moderate heterogeneity, and high heterogeneity, respectively.^[10]^ An *I*^2^ value greater than 50% indicates a significant heterogeneity. A random-effects model was used in the case of significant heterogeneity (*I*^2^ > 50%), otherwise, a fixed-effects model was used.^[11]^ We conducted sensitivity analyses to explore possible explanations for the heterogeneity on the overall pooled estimate and to examine the influence of various exclusion criterions on the overall pooled estimate. We further conducted Begg funnel plots to identify the existence of publication bias. Differences are considered statistically significant at *P* < .05. Statistical analyses were performed by RevMan version 5.3 (Cochrane Collaboration, Oxford, UK), and sensitivity analysis and funnel plots were conducted by STATA STATA 12.0 (StatCorp, College Station,TX, USA).

### 2.7 Trial sequential analysis (TSA)

The same as clinical trial, systematic review and meta-analysis also need to estimate sample size to reduce the risks of random errors and ensure the reliability of results.^[12]^ TSA is a method which could control the risks of type I and type II errors and calculate required information size (RIS) needed by systematic review and meta-analysis.^[13]^ When the cumulative Z curve crosses the trial sequential monitoring boundaries with or without the achievement of RIS, we think the anticipated intervention effect may have been reached and no further trials are needed. If RIS has been reached, but the cumulative Z curve crosses neither the trial sequential monitoring boundaries nor conventional boundaries, we think there is no statistical difference between 2 groups and no more trials are needed. If the cumulative Z curve crosses the futility boundaries, we can also think no difference exists between two groups. However, if the cumulative Z curve does not cross the trial sequential monitoring boundaries, at the same time, the RIS has not been reached, we conclude that more trials are needed.

We adopted a method of constant continuity correction for handing zero-event trials,^[14]^ and added a continuity correction factor of 0.5 to the number of events and non-events in each group.

Two-sided tests, a type I error of 5% and a type II error of 20% (a power of 80%) were used for calculating the RIS. For dichotomous data, incidence in the control was derived from the results of our meta-analysis, and a relative risk reduction or increase was estimated according to the information from related areas.

## 3 Results

### 3.1 Process for included trials

**Figure 1:**
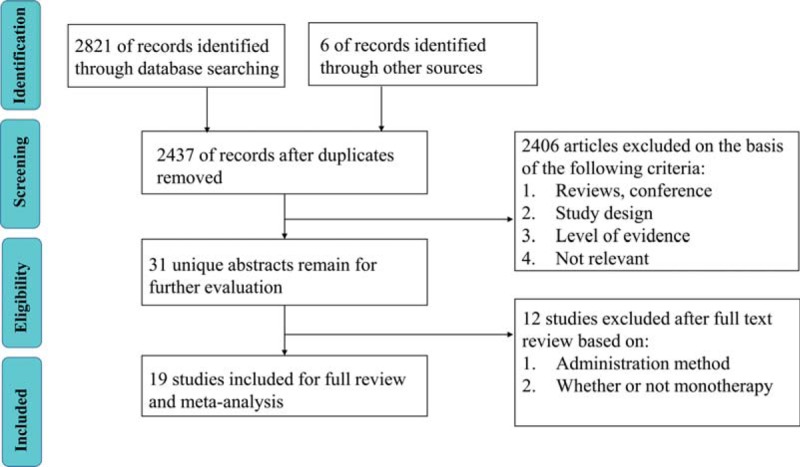
Process for included trials.

As shown in Figure 1, a total of 2827 potentially relevant studies were identified and screened for retrieval. Totally 389 studies were excluded because of duplications and 2400 studies were excluded after the titles and abstracts had been read. Thus 37 studies were assessed for eligibility. Because 15 studies of them included other positive antioxidants, and 3 studies selected oral route for administration, finally 19^[7,15–32]^ RCTs were included in our review.

### 3.2 Characteristics of included trials

The main characteristics of the trials included in our meta-analysis were shown in Table 1. There were totally 3341 critically ill patients of which 1694 participates were in the selenium supplementation group, and 1647 in the control. Diseases in most of studies included SIRS, sepsis, septic shock and multiple organ failure. The doses of selenium supplement on the first day varied from 500 μg to 4000 μg in different studies, and patients in the selenium supplementation group from 13 RCTs received loading bolus on the first day varied from 1000 μg to 4000 μg. In three studies (500 μg/day) and Zimmermann research (1000 μg/day) the patients were given the same dose duration the treatment, while in the rest studies the patients were given a dynamic dose duration the treatment. In the control, patients in 5 RCTs were given a low-dose selenium from 31.6 μg/day to 100 μg/day, and in 7 RCTs were given 0.9% sodium chloride placebo, and in 3 studies were given standard therapy, and in 4 studies such interventions were not reported. The total treating period was reported in 17 trials. Thus, the total dose amount could be calculated by subtracting the control from the selenium supplement group, that is, by subtracting 2050 μg from 28,000 μg. The number of patients in these studies varied from 17 to 1089 and hospital fatality was reported in all studies.

### 3.3 Risk of bias and quality of evidence

**Figure 2:**
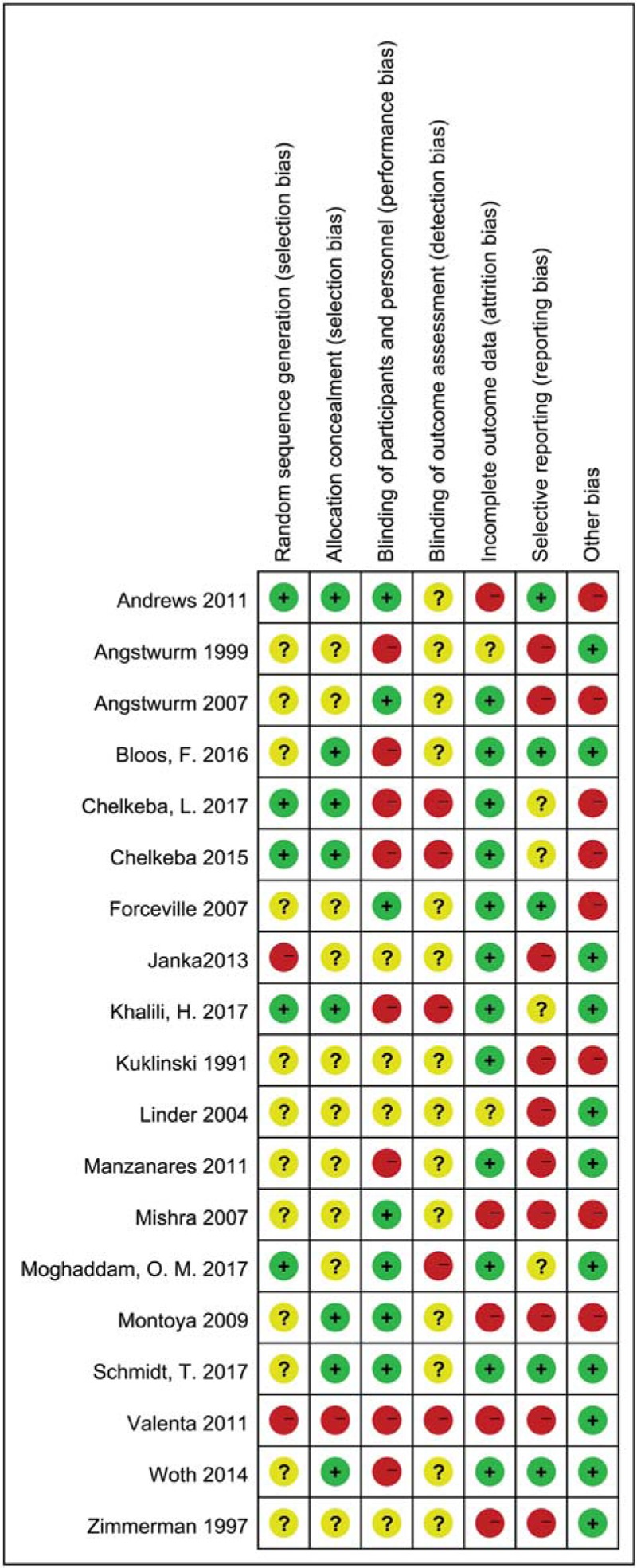
Risk of bias summary.

Figure 2 showed risk of bias in the included trials. The GRADE evidence quality for outcomes was summarized in Table 2.

**Table 2:**
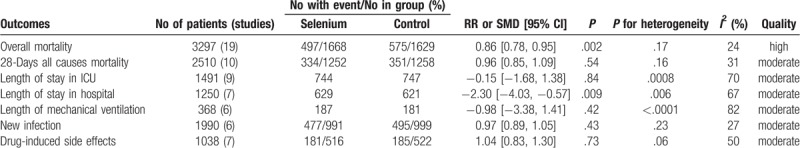
The GRADE evidence quality for outcomes.

### 3.4 Meta-analysis results

#### 3.4.1 Primary end points

##### 3.4.1.1 Overall mortality

**Figure 3:**
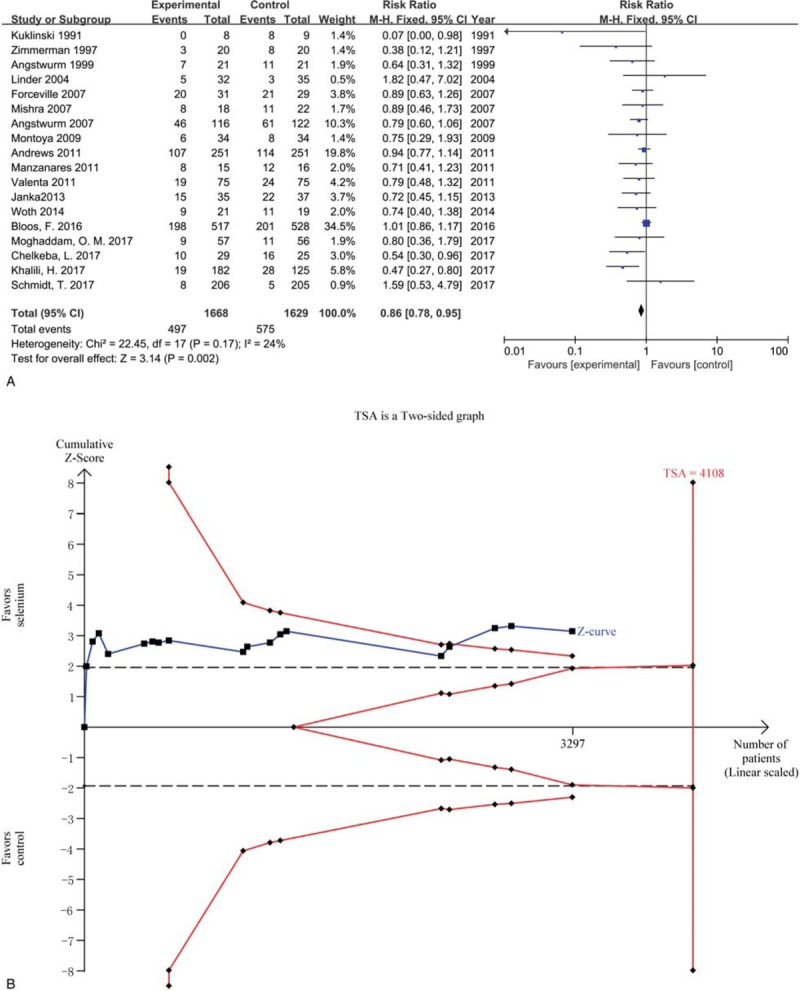
Figure 3A Forest plot for overall mortality. CI = confidence intervals, Fixed = a fixed effects model, M–H = Mantel–Haenszel test, Figure 3B. TSA for overall mortality. TSA = Trial sequential analysis.

We included nineteen trials with 3297 participants reporting overall mortality in 2 groups. The result indicated that selenium supplement could reduce the overall mortality compared with placebo or no intervention in critically ill patients (*RR* 0.86, 95% CI 0.78–0.95, *P* = .002) using a fixed effects model (*I*^2^ = 24%, *P* = .17) (Fig. 3A).

Trial sequential analysis was conducted in the light of overall mortality in the control of 30%, a relative risk reduction in experimental group of 18%, and diversity (D^2^) of 48%. The required information size was 4108 participants, 80.3% of which were accrued in our meta-analysis. The cumulative Z curve (blue line) crossed the trial sequential monitoring boundaries (red inward slash) before the RIS has been reached (Fig. 3B). The TSA-adjusted 95% CI of RR was 0.77 to 0.96.

##### 3.4.1.2 Twenty eight days all causes mortality

**Figure 4:**
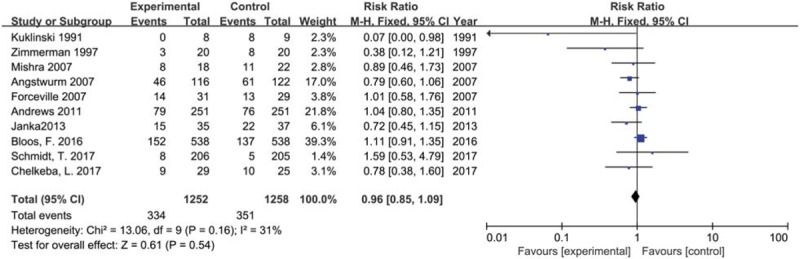
Forest plot for 28-day-all cause mortality. CI = confidence intervals, Fixed = a fixed effects model, M–H = Mantel–Haenszel test.

We included ten trials with 2510 participants reporting 28-day all causes mortality in 2 groups. No significant difference was found between selenium supplement and placebo or no intervention (RR 0.96, 95% CI 0.85 to 1.09, *P* = .54) using a fixed effects model (I^2^ = 31%, *P* = .16) (Fig. 4).

#### 3.4.2 Secondary end points

##### 3.4.2.1 Length of stay in ICU

**Figure 5:**
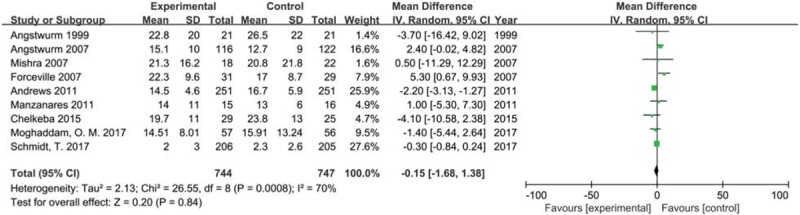
Forest plot for ICU length of stay. CI = confidence intervals, IV = inverse variance, Random = a random effects model.

We included nine trials with 1491 participants reporting length of stay in ICU in 2 groups. The result showed that selenium supplement could not shorten the length of stay compared with placebo or no intervention in critically ill patients (MD −0.15, 95% CI −1.68 to 1.38, *P* = .84) using a random effects model (*I*^2^ = 70%, *P* = .0008) (Fig. 5). The result of sensitivity analysis found that no single study had a significant influence on pooled MD (Additional file).

##### 3.4.2.2 Length of stay in hospital

**Figure 6:**
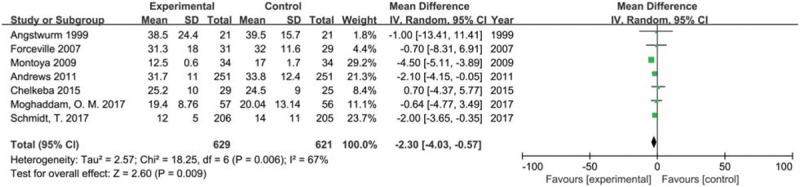
Forest plot for hospital length of stay. CI = confidence intervals, IV = inverse variance, Random = a random effects model.

We included seven trials with 1250 participants reporting length of stay in hospital in 2 groups. The result showed that selenium supplement may shorten the length of stay in hospital compared with placebo or no intervention in critically ill patients (MD −2.30, 95% CI −4.03 to −0.57, *P* = .009) using a random effects model (*I*^2^ = 67%, *P* = .006) (Fig. 6). The result of sensitivity analysis found that no single study had a significant influence on pooled MD (Additional file).

##### 3.4.2.3 Length of mechanical ventilation during follow-up

**Figure 7:**
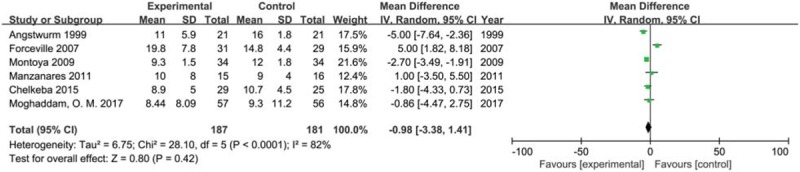
Forest plot for mechanical ventilation time. CI = confidence intervals, IV = inverse variance, Random = a random effects model.

We included 6 trials with 368 participants reporting Length of mechanical ventilation during follow-up in 2 groups. The result showed that selenium supplement could not shorten the length of stay compared with placebo or no intervention in critically ill patients (MD −0.98, 95% CI −3.38 to 1.41, *P* = .42) using a random effects model (*I*^2^ = 82%, *P* < .0001) (Fig. 7). The result of sensitivity analysis found that no single study had a significant influence on pooled MD (Additional file).

##### 3.4.2.4 New infection

**Figure 8:**
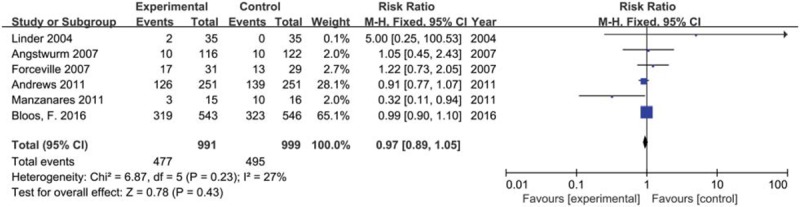
Forest plot for number of infected participants. CI = confidence intervals, Fixed = a fixed effects model, M–H = Mantel–Haenszel test.

We included 6 trials with 1990 participants reporting number of new infected participants in 2 groups. The result showed that selenium supplement could not reduce the number of new infected participants compared with placebo or no intervention in critically ill patients (*RR* 0.97, 95% CI 0.89– 1.05, *P* = .43) using a fixed effects model (*I*^2^ = 27%, *P* = .23) (Fig. 8).

##### 3.4.2.5 Drug-induced side effects

**Figure 9:**
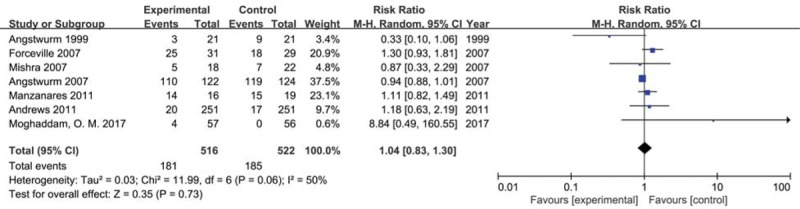
Forest plot for drug-induced side effects. CI = confidence intervals, M-H = Mantel–Haenszel test, Random = a random effects model.

We included seven trials with 1038 participants reporting drug-induced side effects in 2 groups. The result showed that selenium supplement did not increase incidence of drug-induced side effect compared with placebo or no intervention in critically ill patients (*RR* 1.04, 95% CI 0.83– 1.30, *P* = .73) using a random effects model (*I*^2^ = 50%, *P* = .06) (Fig. 9). The result of sensitivity analysis found that no single study had a significant influence on pooled RR (Additional file).

#### 3.4.3 Publication bias

Begg funnel plot showed no publication bias (Additional file).

## 4 Discussion

The pooled results from 19 RCTs using a fixed effects model suggest that selenium supplement could cause decrease in the total mortality in hospital but could not reduce the mortality at day 28. We conduct subgroup analysis such as loading bolus and no loading bolus; high total dose and low total dose; duration≤ 9 days, duration>9 days, and unknown duration, no significant subgroup difference was found. For the complications, results indicate selenium supplement did not increase incidence of drug-induced side effect, but it did not yet cause reduction in the new infections. Data also show that selenium have no influence on the length of stay in ICU or the length of mechanical ventilation. Overall, the clinical heterogeneity is low among these RCTs, and most of the studies are of moderate quality and little differences are found in characteristics of the populations, regimen, and study designs. Sensitivity analysis suggests that the results are relatively stable.

Mortality in critically illness is the primary end point. Our meta-analysis shows that there is significant difference between the selenium supplement group and the control in the total mortality in hospital and the TSA result shows that our conclusion is reliable and no more trials are needed to confirm it, although there is no beneficial effect on the mortality at day 28. Total mortality in our meta-analysis refers to mortality regardless of the follow-up period, however, the longest follow-up period of our included studies is 3 months. According to our results, we suppose that selenium supplement may have a beneficial effect on the clinical outcome of long-term follow-up mortality.

To the best of our knowledge, this is not the first meta-analysis to explore the role of selenium supplement on the outcome of critically ill patients. Our partial results are different from the last meta-analysis.^[9]^ Manzanares et al^[9]^ including 21 studies reported that the use of high-dose selenium supplementation had no beneficial effect on overall mortality and the length of stay in hospital in critically ill patients. They did not use TSA to control the risks of type I and type II errors and calculate RIS. However, TSA was used in present article and the result of TSA demonstrated that our conclusion selenium could cause reduction in overall mortality is reliable and no more studies are needed. In the meta-analysis Manzanares et al^[9]^ selenium as a combined therapy is also included, and the test subgroup difference between selenium as a monotherapy and combined therapy was not significant. Manzanares et al also analyzed mechanical ventilation and the incidence of new infections, and get similar results with our study.

Complications are also assessed. Although selenium supplement is generally regarded as safe and well tolerated in most populations, it should be with cautious that high dose of selenium may lead to toxicity, which is most likely resulted from their prooxidant properties.^[33]^

The meta-analysis has several potential limitations that should be taken into account. Firstly, even though we analyzed selenium supplement in different subgroups, the characteristics of them are different and the effect may be unequal. In the included studies, the characteristics of critically ill patients are not on a unified level, which vary from SIRS to severe multiple injuries. These factors may have a potential influence on our results. Secondly, follow-up varies from 28 days to 12 months, and the outcomes will be uncertain in mutative follow-ups. Thirdly, the route, dose and administration of selenium supplement are varying, so we are not sure to assess the impact of selenium supplement based on clinically meaningful end points. In addition, our study provides additional interesting clues that may be useful for future research on this topic. Remarkably, route of selenium supplement is by continuously intravenous infusion in all studies. Thus, one clue is to focus on route of selenium supplement and to compare the enteral selenium supplement with parenteral selenium supplement to testify the efficacy on critically illness.

In conclusion, the current evidence suggests that the use of selenium could cause reduction in overall mortality and may shorten the length of stay in hospital in critically ill patients, but could not reduce 28-days all causes mortality or shorten length of stay in ICU. Also it has no influence on mechanical ventilation or the incidence of new infections. However, the results should be used carefully because of potential limitations. Further well-designed RCTs on this topic are needed to carry out to provide more evidence to clearly answer the clinical question.

## Author contributions

Hongjun Kang and Feihu Zhou designed the research. Yan Zhao and Xin Hu conducted the research. Li Wang and Zhi Mao analyzed the data. Hongjun Kang and Yan Zhao wrote the manuscript. Feihu Zhou had primary responsibility for the final content. All authors read and approved the final manuscript.

**Conceptualization:** feihu zhou, Hongjun Kang.

**Data curation:**Xin Hu.

**Formal analysis:** Zhi Mao, Li wang.

**Visualization:** Mengmeng Yang, Rrui Yuan.

**Writing – original draft:** Yan Zhao.

## Supplementary Material

Supplemental Digital Content

## References

[1] Chaudhari N Talwar P Parimisetty A A molecular web: endoplasmic reticulum stress, inflammation, and oxidative stress. Front Cell Neurosci 2014;8:213. 25120434 10.3389/fncel.2014.00213 PMC4114208

[2] Liu H Li X Qin F Selenium suppresses oxidative-stress-enhanced vascular smooth muscle cell calcification by inhibiting the activation of the PI3K/AKT and ERK signaling pathways and endoplasmic reticulum stress. J Biol Inorg Chem 2014;19:375–88. 24390545 10.1007/s00775-013-1078-1

[3] Schmidt R Luboeinski T Markart P Alveolar antioxidant status in patients with acute respiratory distress syndrome. Eur Respir J 2004;24:994–9. 15572544 10.1183/09031936.04.00120703

[4] Motoyama T Okamoto K Kukita I Possible role of increased oxidant stress in multiple organ failure after systemic inflammatory response syndrome. Crit Care Med 2003;31:1048–52. 12682471 10.1097/01.CCM.0000055371.27268.36

[5] Marques MB Langouche L Endocrine, metabolic, and morphologic alterations of adipose tissue during critical illness. Crit Care Med 2013;41:317–25. 23135416 10.1097/CCM.0b013e318265f21c

[6] Huet O Cherreau C Nicco C Pivotal role of glutathione depletion in plasma-induced endothelial oxidative stress during sepsis. Crit Care Med 2008;36:2328–34. 18664787 10.1097/CCM.0b013e3181800387

[7] Angstwurm MW Schottdorf J Schopohl J Selenium replacement in patients with severe systemic inflammatory response syndrome improves clinical outcome. Crit Care Med 1999;27:1807–13. 10507602 10.1097/00003246-199909000-00017

[8] Allingstrup M Afshari A Selenium supplementation for critically ill adults. Cochrane Data Syst Rev 2015;Cd003703. 10.1002/14651858.CD003703.pub3 PMC6517228 26214143

[9] Manzanares W Lemieux M Elke G High-dose intravenous selenium does not improve clinical outcomes in the critically ill: a systematic review and meta-analysis. Crit Care (London, England) 2016;20:356. 10.1186/s13054-016-1529-5 PMC5084353 27788688

[10] Higgins JP Thompson SG Deeks JJ Measuring inconsistency in meta-analyses. BMJ (Clin Res Ed) 2003;327:557–60. 10.1136/bmj.327.7414.557 PMC192859 12958120

[11] Armitage P Berry G Matthews JNS Analysing means and proportions. Stat Methods Med Res 2008;83–146. Blackwell Science Ltd.

[12] Brok J Thorlund K Gluud C Trial sequential analysis reveals insufficient information size and potentially false positive results in many meta-analyses. J Clin Epidemiol 2008;61:763–9. 18411040 10.1016/j.jclinepi.2007.10.007

[13] Wetterslev J Thorlund K Brok J Trial sequential analysis may establish when firm evidence is reached in cumulative meta-analysis. J Clin Epidemiol 2008;61:64–75. 18083463 10.1016/j.jclinepi.2007.03.013

[14] Sweeting MJ Sutton AJ Lambert PC What to add to nothing? Use and avoidance of continuity corrections in meta-analysis of sparse data. Stat Med 2004;23:1351–75. 15116347 10.1002/sim.1761

[15] Andrews PJ Avenell A Noble DW Randomised trial of glutamine, selenium, or both, to supplement parenteral nutrition for critically ill patients. BMJ (Clin Res Ed) 2011;342:d1542. 10.1136/bmj.d1542 21415104

[16] Angstwurm MW Engelmann L Zimmermann T Selenium in Intensive Care (SIC): results of a prospective randomized, placebo-controlled, multiple-center study in patients with severe systemic inflammatory response syndrome, sepsis, and septic shock. Crit Care Med 2007;35:118–26. 17095947 10.1097/01.CCM.0000251124.83436.0E

[17] Bloos F Trips E Nierhaus A Effect of sodium selenite administration and procalcitonin-guided therapy on mortality in patients with severe sepsis or septic shock: a randomized clinical trial. JAMA Inter Med 2016;176:1266–76. 10.1001/jamainternmed.2016.2514 27428731

[18] Chelkeba L Ahmadi A Abdollahi M The effect of parenteral selenium on outcomes of mechanically ventilated patients following sepsis: a prospective randomized clinical trial. Ann Intensive Care 2015;5:29. 26429356 10.1186/s13613-015-0071-y PMC4591221

[19] Forceville X Laviolle B Annane D Effects of high doses of selenium, as sodium selenite, in septic shock: a placebo-controlled, randomized, double-blind, phase II study. Crit Care (London, England) 2007;11:R73. 10.1186/cc5960 PMC2206523 17617901

[20] Janka V Ladislav K Jozef F Restoration of antioxidant enzymes in the therapeutic use of selenium in septic patients. Wien Klin Wochenschr 2013;125:316–25. 23644928 10.1007/s00508-013-0371-x

[21] Khalili H Ahl R Cao Y Early selenium treatment for traumatic brain injury: does it improve survival and functional outcome? Injury 2017;48:1922–6. 28711170 10.1016/j.injury.2017.07.005

[22] Woth G Nagy B Merei A The effect of Na-selenite treatment on the oxidative stress-antioxidants balance of multiple organ failure. J Crit Care 2014;29: 883.e887-811. 10.1016/j.jcrc.2014.04.010 24866022

[23] Zimmermann T Albrecht S Kuhne H Selenium administration in patients with sepsis syndrome. A prospective randomized study. Med Klin (Munich)) 1997;92 Suppl 3:3–4. 10.1007/BF03041947 9417494

[24] Kuklinski B Buchner M Schweder R Acute pancreatitis--a free radical disease. Decrease in fatality with sodium selenite (Na2SeO3) therapy. Z Gesamte Inn Med 1991;46:145–9. 1648849

[25] Lindner D Lindner J Baumann G Investigation of antioxidant therapy with sodium selenite in acute pancreatitis. A prospective randomized blind trial. Med Klin (Munich) 2004;99:708–12. 15599680 10.1007/s00063-004-1104-8

[26] Manzanares W Biestro A Torre MH High-dose selenium reduces ventilator-associated pneumonia and illness severity in critically ill patients with systemic inflammation. Intensive Care Med 2011;37:1120–7. 21445641 10.1007/s00134-011-2212-6

[27] Chelkeba L Ahmadi A Abdollahi M The effect of high-dose parenteral sodium selenite in critically ill patients following sepsis: a clinical and mechanistic study. Indian J Crit Care Med 2017;21:287–93. 28584432 10.4103/ijccm.IJCCM_343_16 PMC5455022

[28] Mishra V Baines M Perry SE Effect of selenium supplementation on biochemical markers and outcome in critically ill patients. Clin Nutr) 2007;26:41–50. 17174015 10.1016/j.clnu.2006.10.003

[29] Moghaddam OM Lahiji MN Hassani V Early administration of selenium in patients with acute traumatic brain injury: a randomized double-blinded controlled trial. Indian J Crit Care Med 2017;21:75–9. 28250601 10.4103/ijccm.IJCCM_391_16 PMC5330057

[30] Schmidt T Pargger H Seeberger E Effect of high-dose sodium selenite in cardiac surgery patients: a randomized controlled bi-center trial. Clin Nutr 2017. 10.1016/j.clnu.2017.04.019 28502744

[31] Valenta J Brodska H Drabek T High-dose selenium substitution in sepsis: a prospective randomized clinical trial. Intensive Care Med 2011;37:808–15. 21347869 10.1007/s00134-011-2153-0

[32] Montoya Gc Hl Villalobos Sja Olvera Gc Efecto antiinflamatorio del selenio en pacientes sépticos. Rev Asoc Mex Med Crit y Ter Int 2009;23:199–205.

[33] Heyland DK Selenium supplementation in critically ill patients: can too much of a good thing be a bad thing? Crit Care 2007;11:153. 17692136 10.1186/cc5975 PMC2206521

