# Supplementary material for: The clinical outcomes of selenium supplementation on critically ill patients: A meta-analysis of randomized controlled trials
Source: Medicine (Baltimore). 2019 May 17;98(20):e15473. doi: 10.1097/MD.0000000000015473 (PMC6531101; doi:10.1097/MD.0000000000015473)
Supplement: Supplemental Digital Content [file medi-98-e15473-s001.doc]

**Additional File 2**

**The clinical outcomes of selenium supplementation on critically ill patients: a meta-analysis of randomized controlled trials**

**Figure S1. Sensitivity analysis of ICU length of stay in selenium** **group versus control group.** CI= confidence interval

**Figure S2. Sensitivity analysis of Hospital length of stay in selenium** **group versus control group.** CI= confidence interval

**Figure S3. Sensitivity analysis of mechanical ventilation time in selenium group versus control group.** CI= confidence interval

**Figure S4. Sensitivity analysis of drug-induced side effects in selenium group versus control group.** CI= confidence interval

**Figure S5. begg’s funnel plot of overall mortality.**

z=0.76, Pr>|z|=0.449 (continuity corrected)

**Figure S6. Begg’s funnel plot of 28-day-all cause mortality.**

z=1.61, Pr>|z|=0.107 (continuity corrected)

**Figure S7. Begg’s funnel plot of new infection.**

z=0.00, Pr>|z|=1.000 (continuity corrected)

**Figure S8. Begg’s funnel plot of drug-induced side effects.**

z=0.00, Pr>|z|=1.000 (continuity corrected)
